# Retroposed copies of *RET* gene: a somatically acquired event in medullary thyroid carcinoma

**DOI:** 10.1186/s12920-019-0552-1

**Published:** 2019-07-09

**Authors:** Larissa V. Bim, Fábio C. P. Navarro, Flávia O. F. Valente, José V. Lima-Junior, Rosana Delcelo, Magnus R. Dias-da-Silva, Rui M. B. Maciel, Pedro A. F. Galante, Janete M. Cerutti

**Affiliations:** 1 Laboratório As Bases Genéticas dos Tumores da Tiroide, Universidade Federal de São Paulo, São Paulo, SP Brazil; 2 Centro de Oncologia Molecular, Hospital Sírio-libanês, São Paulo, SP Brazil; 3 Departamento de Bioquímica, Universidade de São Paulo, São Paulo, SP Brazil; 4 Laboratório de Endocrinologia Molecular e Translacional, Universidade Federal de São Paulo, São Paulo, SP Brazil; 5 Departamento de Patologia, Universidade Federal de São Paulo, São Paulo, SP Brazil

**Keywords:** *RET*, Retrocopy, Second hit, Medullary thyroid carcinoma, MTC, G548V, MEN 2

## Abstract

**Background:**

Different pathogenic germline mutations in the *RET o*ncogene are identified in MEN 2, a hereditary syndrome characterized by medullary thyroid carcinoma (MTC) and other endocrine tumors. Although genetic predisposition is recognized, not all *RET* mutation carriers will develop the disease during their lifetime or, likewise, *RET* mutation carriers belonging to the same family may present clinical heterogeneity. It has been suggested that a single germline mutation might not be sufficient for development of MEN 2-associated tumors and a somatic bi-allelic alteration might be required. Here we investigated the presence of somatic second hit mutation in the *RET* gene in MTC.

**Methods:**

We integrated Multiplex Ligation-dependent Probe Amplification (MLPA) and whole exome sequencing (WES) to search for copy number alteration (CNA) in the *RET* gene in MTC samples and medullary thyroid cell lines (TT and MZ-CR-1). We next found reads spanning exon-exon boundaries on *RET*, an indicative of retrocopy. We subsequently searched for *RET* retrocopies in the human reference genome (GRCh37) and in the 1000 Genomes Project data, by looking for reads reporting joined exons in the *RET* locus or distinct genomic regions. To determine *RET* retrocopy specificity and recurrence, DNA isolated from sporadic and MEN 2-associated MTC (*n* = 37), peripheral blood (*n* = 3) and papillary thyroid carcinomas with *RET* fusion (*n* = 10) samples were tested using PCR-sequencing methodology.

**Results:**

Through MLPA we have found evidence of CNA in the *RET* gene in MTC samples and MTC cell lines. WES analysis reinforced the presence of the CNA and hinted for a retroposed copy of *RET* not found in the human reference genome and 1.000 Genomes Project. Extended analysis confirmed the presence of a somatic MTC-related retrocopy of *RET* in both sporadic and hereditary tumors. We further unveiled a recurrent (28%) novel point mutation (p.G548 V) found exclusively in the retrocopy of *RET*. The mutation was also found in cDNA of mutated samples, suggesting it might be functional.

**Conclusion:**

We here report a somatic specific *RET* retroposed copy in MTC samples and cell lines. Our results support the idea that generation of retrocopies in somatic cells is likely to contribute to MTC genesis and progression.

**Electronic supplementary material:**

The online version of this article (10.1186/s12920-019-0552-1) contains supplementary material, which is available to authorized users.

## Background

Medullary thyroid carcinoma (MTC) is a rare form of thyroid cancer originated from the thyroid calcitonin-producing C-cells. MTC can occur in sporadic or hereditary forms. Sporadic MTCs are usually unilateral, characterized by the absence of familial history and not associated with other endocrinopathies [1, 2]. The hereditary form occurs as a part of the multiple endocrine neoplasia type 2 (MEN 2), an autosomal dominantly inherited cancer syndrome. Two clinically distinct subtypes of MEN 2 (MEN 2A and MEN 2B) have been described. MEN 2A and MEN 2B are characterized by the presence MTC, pheochromocytoma (PHEO) and additional endocrinopathies [1].

Germline activating mutation in the REarranged during Transfection (*RET*) gene is pathognomonic in patients with MEN 2A and MEN 2B. Over 80 pathogenic germline variants in the *RET* gene have been described in about 98% of patients with MEN 2 [1, 3].

Although genetic predisposition is recognized and all cells carry the same mutation in a *RET*-carrier patient, not all cells in a given tissue undergo tumorigenesis processes and not all mutation carriers will develop endocrine cancers commonly related to MEN 2 phenotype. In fact, it is generally accepted that most patients with MEN 2A and MEN 2B develop MTC but virtually only 50% of patients develop PHEO during their lifetime. Moreover, at diagnosis, nearly 30% of PHEO in MEN 2 are bilateral and nearly 50% of patients with unilateral disease develop a second PHEO in the contralateral adrenal gland within 10 years [4]. Although the incomplete penetrance can be partially explained by the different nature of the mutations in the *RET* gene or even different genetic backgrounds and environmental factors, they cannot fully explain the organ-specific risks or even variable expressivity in those individuals with a particular *RET* genotype.

Recent works have suggested that MEN 2-associated tumors may require a second hit in the *RET* gene to initiate tumorigenesis, similar to that seen for tumor suppressor genes [5, 6]. The authors have shown that allelic imbalance of the mutant *RET* allele and loss of the wild-type *RET* allele are associated with the genesis and/or progression of MEN 2-associated MTC [7–9] and PHEO [7]. Additionally, it has been stated that the incidence of MEN 2-related PHEO varies throughout different regions of the world, suggesting that the *RET* mutations are not the only determinants of onset age, and that it may be influenced by genetic or environmental modifying factors [10, 11].

Although it is still unclear which phenomenon triggers the second hit event, the most frequent genetic event was duplication of the mutant allele through trisomy of chromosome 10 [7]. Other authors have suggested that additional genetic events, such as somatic *VHL* alterations or chromosomal imbalances may also play a role in the pathogenesis of familial and sporadic MTC cases. These observations led us to consider that the phenotype might be sensitive to concentration of the *RET* gene product.

In search of duplication (second-hit) of the *RET* gene or novel genetic events that might influence development and the progression of MTC, we performed an MLPA assay followed by a whole exome sequencing in a thyroid sample from a MEN 2A patient. Our analysis showed the presence of a higher number of copies of the *RET* gene and hinted for a retrocopy insertion in MTC carcinoma cell lines and MTC tumor tissues, which are lacking in patient-matched blood and normal thyroid samples.

## Methods

### Casistic

This retrospective study analyzed patients who underwent total thyroidectomy at Hospital São Paulo, Universidade Federal de São Paulo. Tissue biopsy was obtained from patients at the time of tumor resection and was frozen in liquid nitrogen. Histology examination confirmed the diagnosis of MTC in 4 patients and C-cell hyperplasia (CCH) in 2 patients. The control group also included peripheral blood sample (*n* = 3) and non-tumoral thyroid tissue (*n* = 3) from patients who developed MTC, peripheral blood from 15 healthy subjects, snap frozen tissue from 11 papillary thyroid carcinomas (PTC) and 4 neuronal tumors.

The validation set included archived formalin-fixed, paraffin-embedded (FFPE) tissue sample from 33 patients with MTC, retrieved from the files of the Department of Pathology of the Universidade Federal de São Paulo. To confirm the diagnosis and to ensure that representative tumor tissue was present in the selected paraffin-embedded section, hematoxylin and eosin (HE) stained slides were reviewed by a pathologist. The most representative tissue block for each tumor was selected.

Patients (*n* = 37) were assumed to have hereditary (*n* = 23) or sporadic MTC (*n* = 14) when no germline mutation in the *RET* gene was found*,* presented a negative family history and no other endocrine disease. *RET* Germline mutation analysis had previously been performed for all patients [12–14] (Additional file 2: Table S1). The study was approved by the Review Boards and Research Ethical Committees of the Universidade Federal de São Paulo,

### Cell lines

The MTC derived cell lines TT and MZ-CRC-1 where kindly donated by Prof. Barry Nelkin from Johns Hopkins University. All cell lines used (TT, MZ-CRC-1 and XTC.U1: Hürthle carcinoma cell) were grown as described in Additional file 2: Table S2. The authenticity of a panel of thyroid cancer cell lines was validated through short tandem repeat (STR) profiling to eliminate concerns of cross contamination.

### DNA isolation

DNA was isolated from fresh-frozen tissues, blood samples and thyroid cell lines using a standard phenol/chloroform method, as previously described [15]. DNA from FFPE samples was isolated from 10 μm-thick sections using the NucleoSpin Tissue kit, according to manufacturer’s instructions (Macherey-Nagel, Dueren, Germany). Only sections with at least 70% of tumor cells were selected for analysis. Tumors samples were carefully dissected to avoid contamination with normal parenchymal and stromal tissues. DNA was quantified using a NanoDrop spectrophotometer (Thermo Fisher Scientific Inc., MA, USA). To prevent RNA contamination, genomic DNA was treated with RNAse A.

### Multiplex ligation-dependent probe amplification (MLPA)

Multiplex ligation-dependent probe amplification (MLPA) has become a standard method for identifying copy number alterations (CNA). All reagents required for MLPA reaction were obtained from MRC-Holland (Amsterdam, The Netherlands). The P169 Hirschsprung-1 SALSA MLPA probemix was chosen as it has probes covering exons 1–21 of the *RET* gene (Additional file 2: Table S3). This assay includes other Hirschsprung-related genes such as: *GDNF* (exons 1–4), *EDN3* (exons 1–5) and *ZFHX1B* (exons 1–10). DNA from MTC fresh frozen tissues (*n* = 4), CCH (*n* = 2) and medullary thyroid cell lines (TT and MZCRC-1) were used for MLPA analysis. DNA isolated from 3 normal thyroid tissues, 3 peripheral blood samples from MTC bearing patients and 3 peripheral blood samples from disease-free patients were used as controls. The reactions were carried out according to manufactures instructions and fragment analysis was performed using ABI 3100 DNA analyzer (PE Applied Biosystems, Foster City, CA). Data analysis was performed by the Coffalyser. Net Software (MRC Holland, Amsterdam, The Netherlands). After intra-sample normalization (on reference probes) and inter-sample normalization (on reference samples), the probe ratios for the target samples were plotted against the mean of the control samples. The intensity of final probe ratios is called Dosage Quocient (DQ). Probes with DQ thresholds at 0.8 and 1.20 are considered to have a normal number of copies; DQ thresholds at < 0.7 and > 1.3 are considerate as deleted or amplified, respectively.

### Next generation sequencing

Whole exome sequencing was performed on DNA extracted from FFPE derived thyroid tissue from a MEN 2A patient. The reaction was performed using the Nextera Rapid-Capture Exome kit (Illumina, San Diego, CA), with 250x coverage (on average) and paired end reads on an Illumina Hiseq2500 system. All procedures were performed by Mendelics Genomic Analysis S. A (São Paulo, Brazil). Reads derived from the exome sequencing were mapped to the human reference genome (GRCh37) using BWA mem (default parameters). We further selected reads mapping to the *RET* locus according to the GENCODE v26 (ENSG00000165731.18). Partial reads were selected using in house developed pipelines that assemble the remaining unaligned portion of overlapping reads. The assembly was then mapped back to the reference genome (version GRCh37) also using BWA (default parameters). We next searched for reads spanning the exon-exon boundaries on *RET* gene, a further indicative of somatic retrocopies, similar to our previous approaches [16].

### *RET* gene retrocopy insertions in human reference genome

To investigate whether *RET* retrocopies are present in normal human genome, the human reference genome sequence (GRCh37) and the *RET* RefSeq [17] transcripts (IDs: NM_020630 and NM_020975) were downloaded from the UCSC Genome Browser [18]. *RET* transcripts were aligned to the human reference genome using BLAT [19] (parameter: -stepSize = 5 –minScore = 0 –minIdentity = 0), and then Perl [20] scripts were developed to search for intronless alignments containing at least 100 nucleotides from two adjacent *RET* exons (which were considered evidence of retrocopy events), similar to our previous approaches [21].

### *RET* gene retrocopy insertions in human populations

To investigate whether *RET* may have retrocopies that are unrepresented in the human reference genome (GRCh37) or even polymorphic retrocopies [22] in the human populations, we applied two strategies previously published by our group [16, 22]. In brief, a set of Perl script was used to analyze the whole genome sequencing data of 2.535 individuals from the 1.000 Genomes Project [23] by searching for reads reporting at least two joined *RET* exons, *i. e.*, we searched for reads reporting genomic intronless *RET* copies (retrocopies). We additionally looked for paired-reads in which some reads (> 3 reads) were mapped on the *RET* locus and their counter-part on distinct genomic regions, suggesting putative genomic insertion points for *RET* retrocopy.

### Investigation and validation of retrocopy in tumor and controls

To investigate whether *RET* retrocopy is a recurrent event in MTC samples, DNA (50 ng) from fresh-frozen tissues and peripheral blood was amplified in a 25-μL PCR reaction containing 5 pmol of each specific primer, 1X PCR Buffer, 0.2 mM of each dNTP, 0.75 mM MgCl*2* and 1 U Taq DNA Polymerase (Invitrogen Corp.). A similar reaction was performed when searching for *RAS* retrocopy. To confirm the identity of PCR products, the long and short bands were excised from 2% agarose gel and purified using the Illustra GFX PCR purification kit (GE Healthcare Corp., Piscataway, NJ, USA), according to manufacturer’s instructions. Purified bands were submitted to direct sequencing using the Big DyeTerminator Cycle Sequencing Ready Reaction Kit and the ABI 3100 sequencer, according to manufacturer’s instructions (PE Applied Biosystems, Foster City, CA). For the validation step, DNA (150 ng) isolated from paraffin-embedded samples was amplified in a 50-μL PCR reaction containing 10 pmol of each specific primer, 1X PCR Buffer, 0.2 mM of each dNTP, 1.5 mM MgCl*2* and 2 U Taq DNA polymerase. Each sample was sequenced at least twice and in both directions. Primers for all exons were designed based on the reference genome (GRCh37) using Primer3 software [24]. Primers sequence and product sizes are shown in Additional file 2: Table S4.

### RNA isolation, cDNA synthesis and sequencing

In order to determine whether the retrocopy is expressed, total RNA was extracted from 10 μm-tick FFPE MTC samples using Recover All Total Nucleic Acid Isolation kit (Invitrogen Corp., California, USA), according to manufactures instructions. Total RNA was quantified by NanoDrop 200c spectrophotometer (Thermo Fisher Scientific Inc.). About 500 ng of total RNA was treated with DNAse and reversed-transcribed into cDNA with oligo-dT12–18 and random primers using a Superscript III reverse transcriptase kit (Thermo Fisher Scientific Inc.). Primer set 8 (Additional file 2: Table S4) was used to amplify *RET* target sequence using 1 μL of cDNA. Purified products were sequenced using BigDye terminator on an ABI 3730 DNA Analyzer (PE Applied Biosystems).

### Statistical analysis

Statistical analysis was performed using StatView 4.5 and GraphPad Prism 5.01 software. Correlation between genetic events and clinical and pathological features were performed using Fisher’s exact test (*n* < 20 per group) and Chi-squared test for categorical data. As all groups were considered normal by the Shapiro-Wilk test, Student’s *t*-test was used for quantitative data. Statistical significance was considered when *p*-value < 0.05.

## Results

### Assessing somatic *RET* amplification through MLPA-based analysis

In search of duplication events (second-hit) in the *RET* gene that could be related to MTC pathogenesis, we performed a Multiplex ligation-dependent probe amplification analysis. The multiplex MLPA-based strategy presented in this study was originally developed to detect germline copy number variation (CNV) of 4 genes related to Hirschsprung disease, including the *RET* gene (exons 1–21) (Additional file 2: Table S3).

**Fig. 1:**
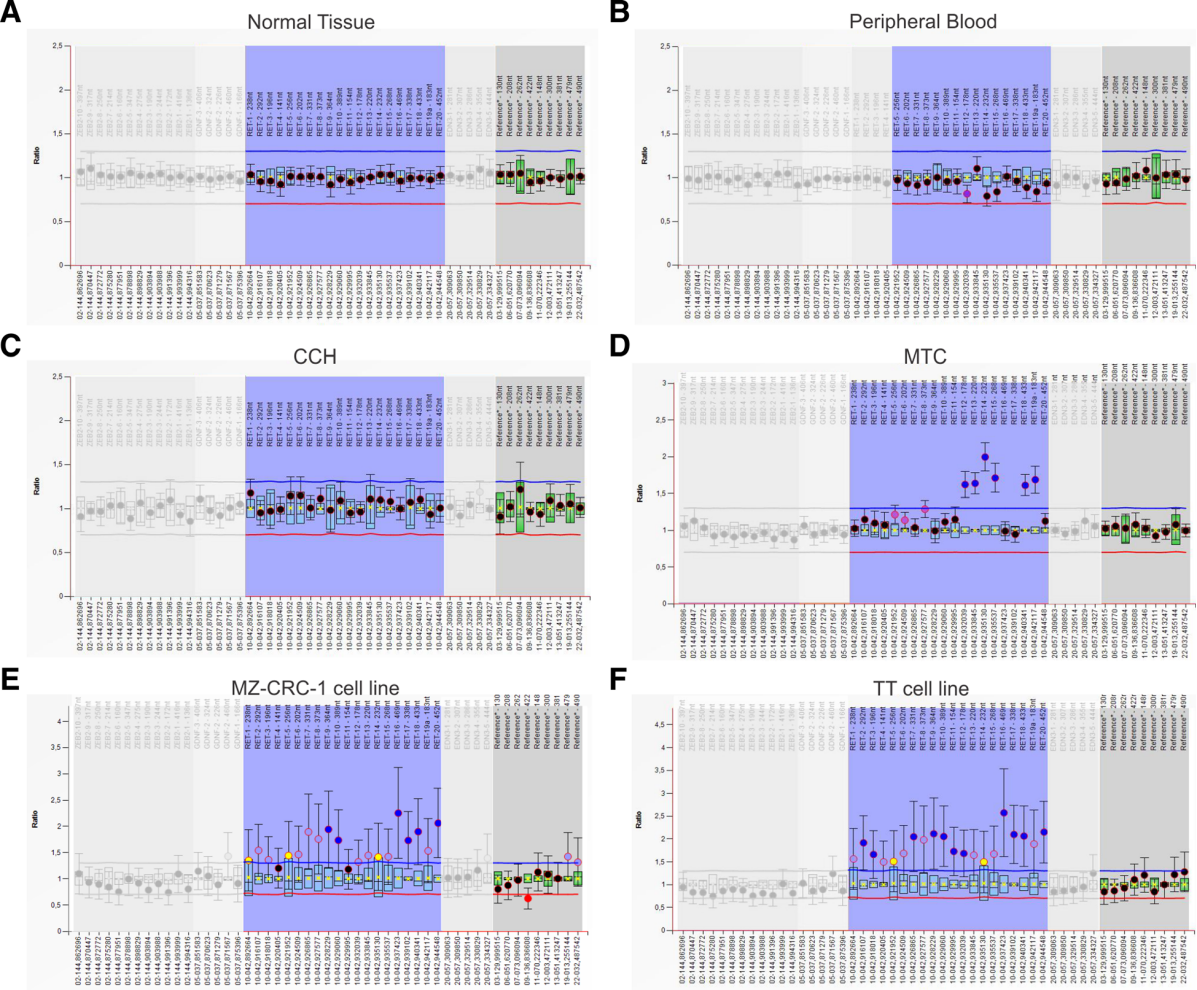
Chart obtained by Coffalyzer analysis showing relative probe ratio values from MLPA results of *RET* gene analysis. The upper and lower bars represent the threshold values of normality. MLPA shows normal ratio for *RET* exons 1–20 in (**a**) normal thyroid tissue (**b**) peripheral blood and (**c**) C-cell hyperplasia, corroborating normal copy numer. Bullets located upper the duplication cut-off line (upper bar) in the ratio chart were identified in (**d**) Medullary Thyroid Carcinoma (MTC) and MTC- derived cell lines (E) MZ-CRC-1 cell line and (**f**) TT cell line, which denoted somatic duplication

The somatic pattern of CNV, therefore named (CNA), showed by the MLPA analysis was complex. As expected, the region target by *RET* probes had two copies in all normal samples (blood sample and non-tumoral thyroid tissue from MTC patients and blood samples from healthy individuals), and also two copies in CCH **(**Fig. 1a-c**).** On the other hand, the *RET* gene was partially duplicated in MTC samples (Fig. 1d), whereas it was fully duplicated in two medullary thyroid cell lines (TT and MZ-CR-1) (Fig. 1 e-f).

The duplicated DNA copies within one sample may vary from each other in length and sequence, as *RET* gene may present mutations or SNPs in heterozygosis located within the hybridization probe region and, therefore, it can affect the affinity of the MLPA probes. No alteration in the gene dosage was observed in the other three genes related to Hirschsprung disease *(GDNF*, *EDN3* and *ZFHX1B*) in all controls, thyroid samples and thyroid cell lines.

### Somatic retrocopies of *RET* gene in a MEN 2A patient

Next, in order to refine the mechanism that generates the *RET* CNA identified by MLPA in MTC samples; we used a next-generation sequencing (NGS) strategy (whole exome sequencing, WES). Particularly, NGS poses an orthogonal method to understand and validate the existence of *RET* duplications.

**Fig. 2:**
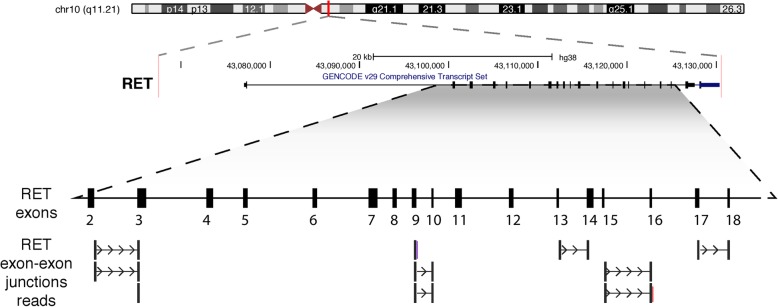
Genomic representation of whole exome sequencing data showing reads spanning exon-exon boudaries. We found reads spanning 10 exon-exon boundaries, from exon 2 to exon 18 (considering transcripts with the highest number of exons annotated). Representation based on UCSC Genome browser plot for the *RET* locus

By analyzing the *RET* locus on chromosome 10q11.21, we acknowledged that there were differences in the coverage of each gene located in this region. A higher coverage of reads was observed in a region containing the RET gene. The immediately upstream and downstream regions exhibited similar levels of coverage, which was lower than that observed for *RET* gene (Additional file 1: Figure S1). This data supports that this region in which *RET* gene is located may carry a duplication. Additionally, the high coverage of reads on *RET* exons and, intriguingly, some reads spanning exon-exon boundaries of the *RET* gene suggested a intronless copy of the gene (Additional file 1: Figure S1). As previously described [25], genomic sequencing with reads spanning exon boundaries suggest the occurrence of a somatic retroduplication event (i.e., an intronless copy of the gene). In a next step, we selected only those reads potentially supporting the *RET* retroduplication event (hereinafter called retrocopy). We detected 10 reads supporting 5 exon-exon boundaries of *RET*, exons 2–3, 9–10, 13–14, 14–15 and 17–18 (Fig. 2). Thus, these data gave us ground to look for undescribed retrotransposed copies of the *RET* gene in humans and particularly, in MTC samples. Importantly, we observed that both extremities of the *RET* gene were not well covered by the WES, therefore, our analysis is agnostic to identify the genomic location where the somatic *RET* retrocopies are inserted (Additional file 1: Figure S1) in MTC samples.

### Assessment of germline *RET* copy-number variants caused by retrotransposition (retroCNVs) in humans

Given that some retrocopies are polymorphic (retroCNVs) in human population and are absent from the reference genome, we next investigated the existence of an undescribed retroCNV of *RET* in the human population. First, we applied a bioinformatic pipeline designed to find miss- or unannotated retrocopies absent in the human reference genome (GRCh37), but present in some genomes from the 1000 genome projects, i.e. retroCNVs (see Material and Methods for details). No evidence was found of *RET* as a retroCNV in the 2.535 genomes from the 1.000 Genomes Project [23].

### Experimental validation of retrocopies of *RET*

**Fig. 3:**
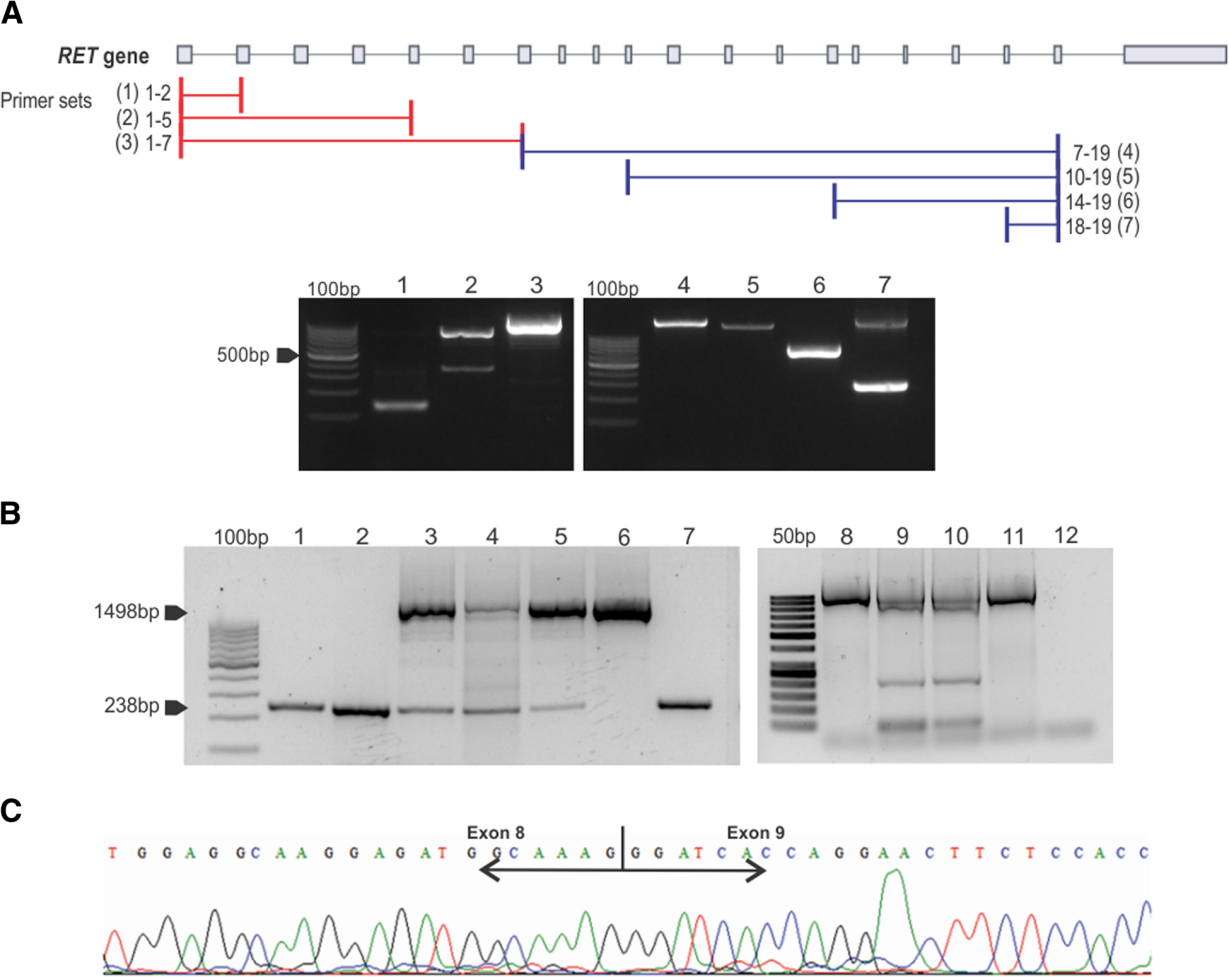
Detection of Somatic retrocopies. (a) Schematic figure representing the RET parental gene and the primer sets (1–7) used to amplify the RET retrocopy. The line represents the non conding sequence (introns and UTR) and the bars represent the exons. The agarose gel shows bands at the expected sizes for intronless copy for all primer sets tested, indicating the presence of a retrocopy of *RET* in genomic DNA isolated from MTC. As expected, the band corresponding to the intron-containing fragment (parental copy) was observed only for primer set 7 (1868pb). The additional band indentified in line 2 of primer set 2 is an inespecific PCR product (Additional file 2 :Table S2). (**b**) Representative PCR analysis performed in MTC and control samples using primers located at exons 7 and 9 (primer set 8, Additional file 2: Table S2) of *RET* gene. The upper band corresponds to the expected PCR product (1498 bp) for *RET* parental gene and the lower band (238 bp) corresponds to the retrocopy (intronless copy). The lower band is seen only in tumor samples (Lines 1–5) and MTC derived cell lines (lines 9 and 10). Lines 1 and 2 were FFPE derived sample and lines 3–5 are fresh frozen tissue drived samples. The lower band was not seen in peripheral blood DNA from patients (line 6), non-MTC cell line (line 11) and MTC cell line obtained from ATCC (third passage). Positive (line 7) and Negative (line 12) controls were used. (**c**) Sequencing analysis of the retrocopy band (238 bp) confirming exon-exon junction

In order to validate our findings based on the NGS data, 7 primer sets were designed to span exon–exon junctions of *RET* gene and used to amplify genomic DNA isolated from fresh frozen MTC by PCR (Fig. 3a). As in the *RET* gene (parental copy) the introns are present and their lengths vary widely, the PCR reactions would generate products that are larger than 1400 bp up to several thousand bp long (1498 bp-34,031 bp). Consequently, it may result in partial product and thus no amplification. If a retrocopy is present, then PCR products should result in one short intron-less amplified sequence. The predicted sizes for the parental gene and for the retrocopy are shown in Additional file 2 : Table S4.

As expected, in this experimental validation, single bands were observed for primer sets 1 to 7 (Fig. 3a). The PCR bands corresponded to the predicted size for an intron-less copy. The products were sequenced and gene identity was confirmed. PCR reactions failed to amplify the longest expected products for *RET* parental copy.

### Detection of cancer-specific gene retrocopy insertions

To determine whether the retrocopy is a cancer-specific somatic variant, we analyzed 3 pairs of tumor and blood samples. We used primer set 8, which encompass the region between exons 7 to 9, as this region is recurrently found amplified in the MLPA assay and its parental copy (1498 bp) and retrocopy (238 bp) could be straightforwardly amplified in genomic DNA isolated from frozen MTC samples. Using this set of primers, one long sequence containing the intron and one short intron-less sequence were observed. The parental *RET* gene (1498 bp band) was present in both tumor and blood samples, while the retrocopy band (238 bp) was observed only in DNA isolated from MTC samples, indicating that it is a cancer-specific event. The analysis was expanded to include additional 33 MTC samples, two MTC cell lines (TT and MZ-CR-1), 2 CCH and 15 DNA isolated from disease-free peripheral blood. The *RET* parental copy was observed in high molecular weight DNA isolated from all non-FFPE samples. As the DNA isolated from FFPE sections is degraded, the 1498 bp was not observed in the DNA isolated from the 33 MTC samples conserved in paraffin-embedded sections. The retrocopy band was observed in all MTC samples (23 hereditary and 14 sporadic) and two MTC cell lines, while it was absent in peripheral blood and CCH **(**Fig. 3b**)**. Sequencing of the retrocopy band showed the perfect junction between exons 7–8-9 **(**Fig. 3c**)**.

Interestingly, when we tested a recently acquired MTC cell line from the ATCC cell bank (TT), the retrocopy band was not observed **(line 8;** Fig. 3b**)**, suggesting that this probably occurs as a secondary genetic event involved in the progression of MTC rather than its genesis. In fact the TT and MZ-CRC-1 have been continuously propagated. The XTC.U1 cell line was used as a negative control.

### *RET* retrocopy is an event related to MTC pathogenesis

**Fig. 4:**
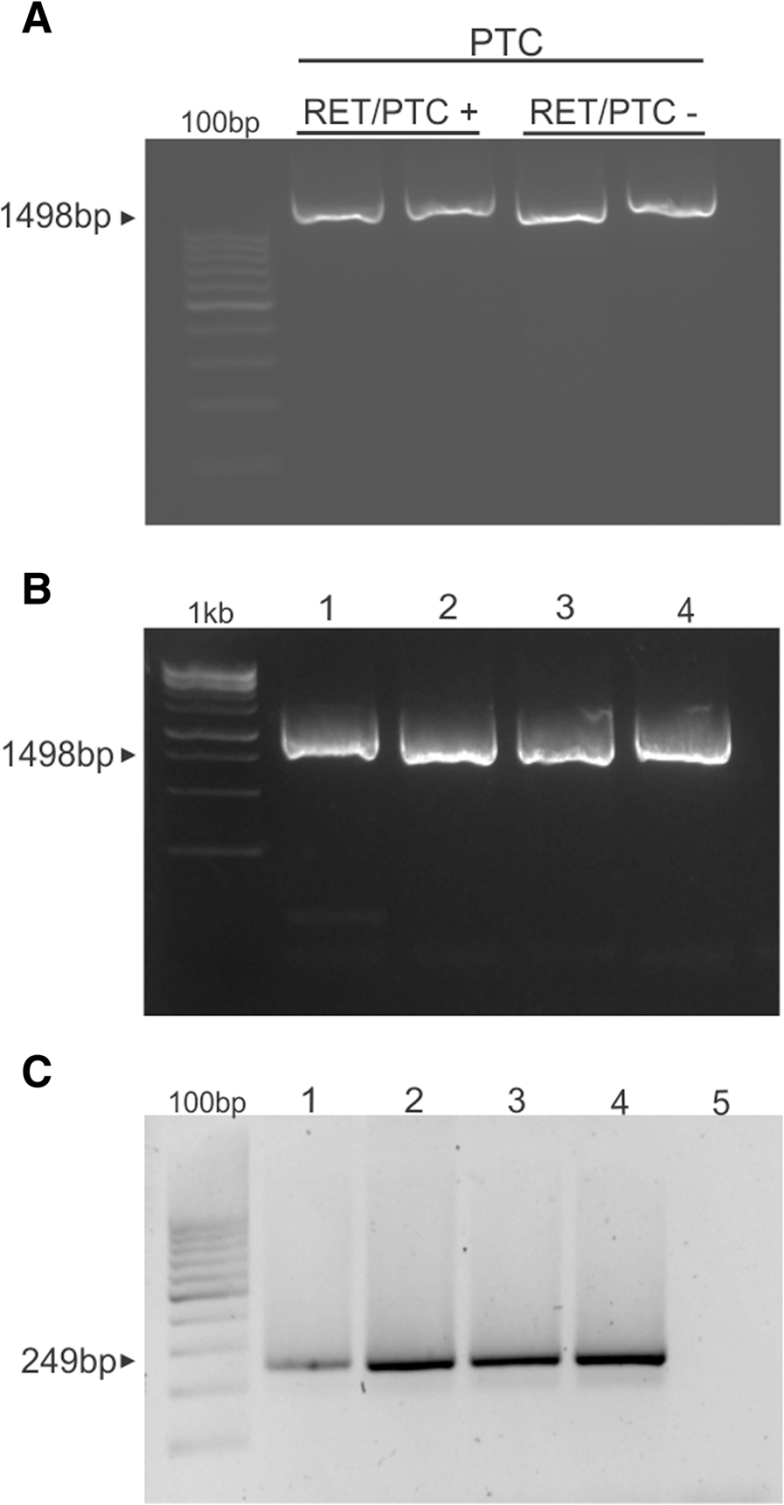
Detection of somatic retrocopies in (**a**) PTC samples positive or negative for the *RET/PTC* rearrangent and (**b**) neuroblastoma (line 1), craniopharyngioma (line 2), pilocytic astrocytoma (line 3) and ependymoma (line 4). (**c**) Amplification of *KRASP1* (processed pseudogene) in MTC (line 1) peripheral blood (line 2) normal thyroid tissue (line 3) positive control (line 4) and negative control (line 5)

We next sought to investigate whether *RET* retrocopy could arise in PTC with *RET* fusions or if they are specifically associated with the pathogenesis of MTC. Therefore, PTC samples (*n* = 11), previously characterized for the presence/absence of *RET/PTC* rearrangements were tested for the presence of *RET* retrocopy. Only the parental sequence containing the introns was observed in *RET/PTC*-positive and negative PTCs **(**Fig. 4a**)**. These findings suggest that the presence of the retrocopy is not necessarily associated with the presence of genetic events involving *RET* gene. We next tested tumors of neuronal origin (*n* = 4), which, similarly to MTC, are derived from neural crest cells. Only the parental copy was observed in the neuronal origin tumors **(**Fig. 4b**)**.

### KRASP1: KRAS retrocopy

Similarly to *RET*, mutations in the *RAS* gene were previously associated with sporadic MTC development [26]. As *KRAS* has a retrocopy (also named as processed pseudogene) called *KRASP1* fixed in the human genomes [27], we designed primers in the exons 1 and 2 of *KRAS* to investigate the presence of *KRASP1* in DNA isolated from peripheral blood, normal thyroid tissue and MTC samples. This strategy would allow the identification of the fixed retrocopy of *KRASP1*, confirming our validation strategy. As expected, *KRASP1* was found in DNA isolated from blood, normal thyroid and MTC **(**Fig. 4c**)**. All together, these findings confirm our validation strategy and highlight a possible role of the *RET* retrocopy in the development and/or progression of MTC.

### P.G548V mutation found in the *RET* retrocopy might be functional

**Fig. 5:**
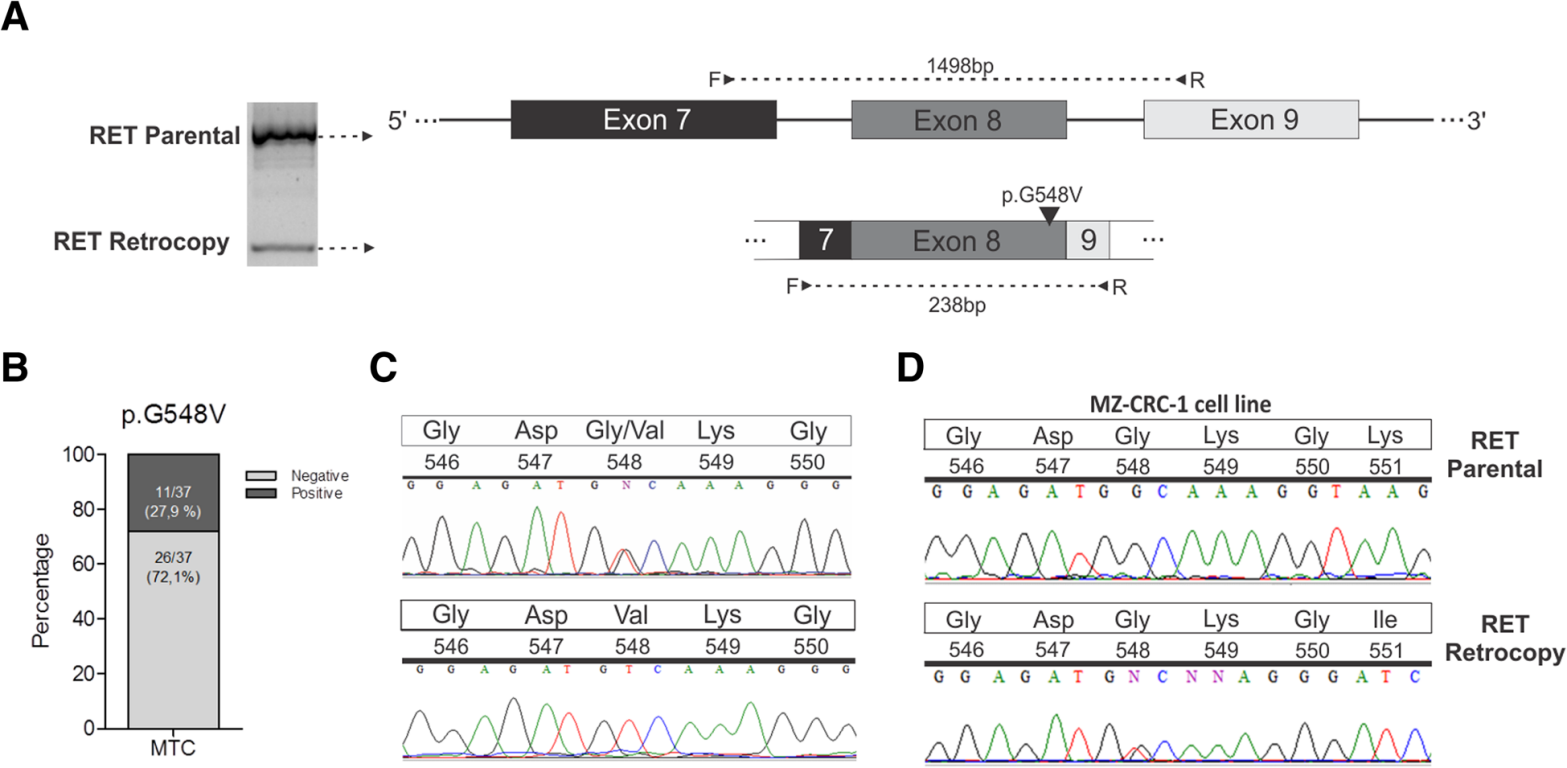
(**a**) Schematic representation of exons 7 to 9 of the *RET* parental gene and retrocopy. Rectangles denote exons, full line denotes introns. The arrow marks the location of the exon 8 p.G548V mutation. The horizontal arrows indicate the location and orientation of primers used. (**b**) Prevalence of the p.G548V mutation on MTC samples, showing that 27,9% of all MTC tested samples are positive. (**c**) Representative electropherogram showing the presence of the mutation both in heterozygosis and homozygosis. The mutations consist of a genomic 1644G > T change. (**d**) Sequencing of DNA amplification from parental *RET* gene and its retrocopy from MZ-CRC-1 cell line showing the p.G548V mutation present only in the retrocopy

Direct sequencing of both PCR bands (obtained with primer set 8) revealed a recurrent point mutation in the exon 8 of the *RET* gene present only in the retrocopy **(**Fig. 5a**)**. The GGC > GTC mutation at position 1644, which leads to a p.G548V substitution, was found in nearly 28% (11/37) of MTC samples (Fig. 5b). Interestingly, the mutation was found in homo and heterozygosis (Fig. 5c), suggesting that more than a copy of the *RET* retrocopy may be present. Among the MTC derived cell lines tested, the retrocopy identified in the MZ-CRC-1 was positive for the p.G548V mutation **(**Fig. 5d**)**.

In order to determine if the retrocopy is expressed, we performed RT-PCR experiments and the PCR products were purified and sequenced. We found evidences of expression, as the p.G548V RET mutation was found in cDNA synthetized from 3 MTCs harboring the retrocopy carrying p.G548V mutation.

### No correlation was found between *RET* p.G548V and clinical and pathological features

The presence of the p.G548V mutation did not correlate with clinical and pathological features (gender, tumor size, presence of metastasis, age of diagnostic) or presence or absence of other common mutations in the *RET* parental gene (sporadic or familial cases) (Table 1).

**Table 1:** Clinical and pathological features of MTC positive and negative for *RET* p.G548V mutation

	MTC (*n* = 37)	p.G548 V Negative (*n* = 26)	p.G548 V Positive (*n* = 11)	*P*-value
Gender				
Male	20	12/26 (46%)	8/11 (73%)	0.1691
Female	17	14/26 (54%)	3/11 (27%)	
Sporadic	14	9/26 (35%)	5/11 (45%)	0.7130
Familial	23	17/26 (65%)	6/11 (55%)	
MEN 2A	21	15/17 (88%)	6/6 (100%)	1.000
MEN 2B	2	2/17 (12%)	0/6 (0%)	1.000
Homozygosis	5	–	5/11 (45%)	–
Heterozygosis	6	–	6/11 (55%)	
Age of Diagnostics (mean ± SD)		38.17 ± 14.28	45.82 ± 18.61	0.1903
Metastasis				
Yes	19	15/26 (57,7%)	4/11 (36,4%)	0.2252
No	11	6/26 (23%)	5/11 (45,5%)	
*RET* mutations				
Negative	11	8/26 (30%)	3/11 (28%)	1.000
Positive^a^	26	18/26 (70%)	8/11 (72%)	
p.G533C	9	6/18 (33%)	3/8 (38%)	1.000
p.C634_	6	4/18 (27%)	1/8 (12%)	0.6279
p.C634Y/Y791F	7	5/18 (28%)	2/8 (25%)	1.000
p.M918 T	4	2/18 (12%)	2/8 (25%)	0.5633

^a^Somatic (*n* = 3) and Germline (*n* = 23). For details, see supplementary Table 1

## Discussion

In the last decade, we have witnessed a massive number of publications of whole genome sequencing (WGS) and whole exome sequencing (WES) projects, along with analysis of gene duplication events as a mechanism of new gene emergence. Therefore, the early notions that gene duplications provide an important reservoir for new genes and hence human evolution, phenotypic adaptation and human diseases, have now been universally confirmed [28–31]. Analogously, somatic gene duplication (i.e.*,* somatic copy number alteration) is widespread in human cancers and has been predicted to drive tumorigenesis [32, 33].

In search of a second-hit on the *RET* gene that might influence development and progression of MTC, we here conducted an MLPA assay followed by WES analysis in a thyroid sample from a MEN 2A patient and found evidences of *RET* retroduplication. Through the MLPA technique we found different patterns of *RET* genomic duplication in MTC samples and MTC-derived cell lines, while no alteration of gene dosage was observed in DNA isolated from controls. The WES data showed a higher coverage of reads in a region containing the *RET* gene, while regions immediately upstream and downstream exhibited lower and similar levels of coverage, reinforcing that this genomic region may carry a duplication. When the *RET* reads from WES were mapped to the human reference genome, exon-exon junctions were identified, suggesting that the molecular mechanism of gene duplication in *RET* involved RNA-mediated gene retroposition. When gene duplication arises by means of reverse transcription of an mRNA and is integrated in the genome, it is referred to as a retrocopy. As *bona fide* retrocopies differ from their parental genes due to the presence of exon-exon junctions and a genomic position different from the *locus* of the parental gene, we searched for the insertion points of the *RET* retrocopy. However, although exome data increases the coverage of exon extremities when compared to WGS, our analysis failed to find the insertion points of the retrocopy.

We next sought to exclude inter-individual genetic variation. When gene duplication processes have a germline origin, the novel gene duplicates have the chance to spread in the population. When fixed retrocopies are found in the population as segregating variants, the retrogenes are named RetroCNV (copy number variation). We, therefore, assessed the presence of *RET* retroCNVs in humans. No evidence of polymorphic *RET* retrocopies in the human reference genome or in the genomes from the 1.000 Genomes Project was found. The experimental validation in an MTC sample, using a set of primers spanning exon-exon junctions, provided proof-of-concept of the presence of a retrocopy of the *RET* gene, advocating a somatic origin of the *RET* retrocopies in our set of MCT samples.

To further ascertain if this retrocopy is MTC-specific, we assessed if it was present in DNA isolated from normal thyroid and peripheral blood from MEN 2A and MEN 2B patients. The parental *RET* copy was present in both tumor and blood samples, while the *RET* retrocopy was MTC-specific. PCR amplification confirmed the presence of retrocopy in all sporadic and hereditary MTC tested. These findings, in association with the screening of PTC samples with *RET/PTC* fusion and neuronal tumors, suggests that this somatic event might be a tissue-specific genetic event and occurs independently of the *RET* mutational status. Interestingly, when a recently acquired MTC cell lineage was tested on a low passage, the retrocopy band was not observed, suggesting this secondary event may occur during tumor progression.

Several somatic genetic events have been described in chromosome 10 and MEN 2-related tumors, showing that this is a region prone to genetic instability. Allelic imbalance between mutant and wild-type *RET* in MEN 2-related tumors have been described in the literature [7, 9, 34], and may represent a decisive step on the genesis of these tumors [7, 9, 35]. A recent work on this field found a high rate of chromosome 10 aneuploidy in sporadic MTC with *RET* somatic mutation. Additionally *RET* gene amplification was found in MEN 2A-associated MTC. The authors suggested that *RET* mutations might confer a higher rate of genetic instability, enabling additional genetic events [8]. However, so far, none of these studies explored the molecular mechanisms involved.

Previous analyses indicate that genes highly expressed are more likely to give rise to novel retrocopies in the soma [31]. In fact, nearly 2.6% of lung and colorectal cancers have somatically acquired retrocopies, and 63% of their parental genes are among the most expressed for these tumor types [36]. As *RET* is highly expressed in MTC, its mRNA has a high likelihood to undergo retrotransposition and to give rise to retrocopies. It has been suggested that these retrocopies might have some implications in terms of cancer progression, as they could give rise to novel, potentially expressed genes [37, 38].

Although most of the retrocopies across the genome are complete or near complete and may be inserted into a genomic background permissive to their expression, many of these new gene retrocopies may be inactive due to missing promoters, frameshifts, and truncation [30, 39]. Accordingly, the concept of retrocopy has been revisited. Transcribed retrocopies were defined as retrogenes, while those that are not functionally active were defined as pseudogenes [39, 40]. Retrogenes, which are transcribed retrocopies, can have a widespread transcription or be tissue or cell type specific or even specific to particular tumors [16, 36, 37, 41]. Retrogenes could alter parental gene expression pattern, either by increasing the levels of an already expressed gene, or by negative regulation of the parental gene through different mechanisms [38]. They may work regulating parental gene expression serving as lncRNA [29, 38, 42], by competing with the parental gene for miRNA sites [29, 38]; they can also be competitive inhibitors of translation of parental transcripts [38, 43] or affect their stability [38, 44].

Here, we unveiled a recurrent (28%) novel point mutation (p.G548V) found exclusively in the retrocopy of *RET*. The only evidence in the literature or databases of a mutation on codon 548 of *RET* has been found in the ExAC database [45], in only one patient of African origin reported with MEN 2 syndrome, but with a different substitution (G548S).

To define whether the retrocopy was functional, the cDNA of MTC samples harboring the p.G548V mutation were sequenced. Using such strategy, we were able to confirm that the retrocopy is expressed as the cDNA was positive for the RET p.G548V mutation, although its role in the pathogenesis and/or progression of MTC is still unknown. So far, basic in silico analysis of the mutation presumes a more damaging effect. PolyPhen-2 [46] has rated it as Probably Damaging with a score of 0.999 (sensitivity: 0.14; specificity: 0.99) and SIFT [47] has rated it as Damaging with a score of 0.034, but PROVEAN [47] predicts it as Natural with a score of − 2.04.

Although many studies involving MTC samples and Next Generation Sequencing (NGS) techniques have been published in the last years, none of them has reported a *RET* retrocopy. It is important to note, however, that most of them are targeted panels [48–50] or exome sequencing studies [26, 51–53] that did not focus on detecting a second hit on the *RET* gene or even additional events that are associated with MTC progression.

By testing *KRASP1* pseudogene, we have obtained evidences that our strategy is accurate to identify retrocopies and that the somatic event described in MTC does not represent “noise” but is potentially associated with progression of MTC.

## Conclusion

In summary, as far as we know, this is the first study that reported a *RET* retrocopy. Our findings suggest that it is a somatic, MTC related retroposed copy of *RET* with a high prevalence of a point mutation in exon 8 (p.G548 V). Generation of retrocopies in somatic cells may have important implications for tumor genesis and progression. To determine the functional implications of the retrocopy and the mechanism by which *RET* mRNA is retroposed in the genome warrants further work.

## Additional files

Additional file 1: **Figure S1.** Visual representation of coverage and read alignment of the WES data. The upper panel shows the coverage and read pileup representation of a section of chromosome 10q. It is possible to notice a region with higher number of reads aligned (as delimited by the dotted box) than the regions immediately upstream and downstream of the indicated region. The bottom panel shows a zoom up of the highlighted region, that contains the *RET* gene. Coverage and read pileup for all exons of the *RET* gene are depicted. No reads were aligned in introns of *RET* gene. (TIF 34059 kb)

Additional file 2: **Table S1.** *RET* analysis in MTC samples used in this study. **Table S2.** Cell lines used in this study **.Table S3.** Probe specification for MLPA P169 Hirschsprung-1 assay as designed by MRC-Holland (Amsterdam, The Netherlands). **Table S4.** Primer design and expected PCR product size for parental copy and Retrocopy. (DOCX 53 kb)

## Data Availability

The WES dataset analyzed during the current study is not publicly available due the fact that a full report including additional data will be available in due course, however it is available from the corresponding author upon reasonable request and with permission of Rui M.B.Maciel. All other data generated or analyzed during this study are included in this published article [and its supplementary information files].

## Abbreviations

CCH: C-Cell Hyperplasia
CNA: Copy Number Alteration
CNV: Copy Number Variation
FFPE: Formalin Fixed Paraffin Embedded
KRAS: Kirsten rat sarcoma viral oncogene homolog
KRASP1: KRAS Pseudogene 1
MEN 2: Multiple Endocrine Neoplasia type 2
MLPA: Multiplex Ligation-Dependent Probe Amplification
MTC: Medullary Thyroid Carcinoma
NGS: Next Generation Sequencing
PHEO: Pheochromocytoma
PTC: Papillary Thyroid Cancer
RET: REarranged during Transfection gene
WES: Whole Exome Sequencing
