# Supplementary figures and images for: Retroposed copies of RET gene: a somatically acquired event in medullary thyroid carcinoma
Source: BMC Med Genomics. 2019 Jul 9;12:104. doi: 10.1186/s12920-019-0552-1 (PMC6617568; doi:10.1186/s12920-019-0552-1)

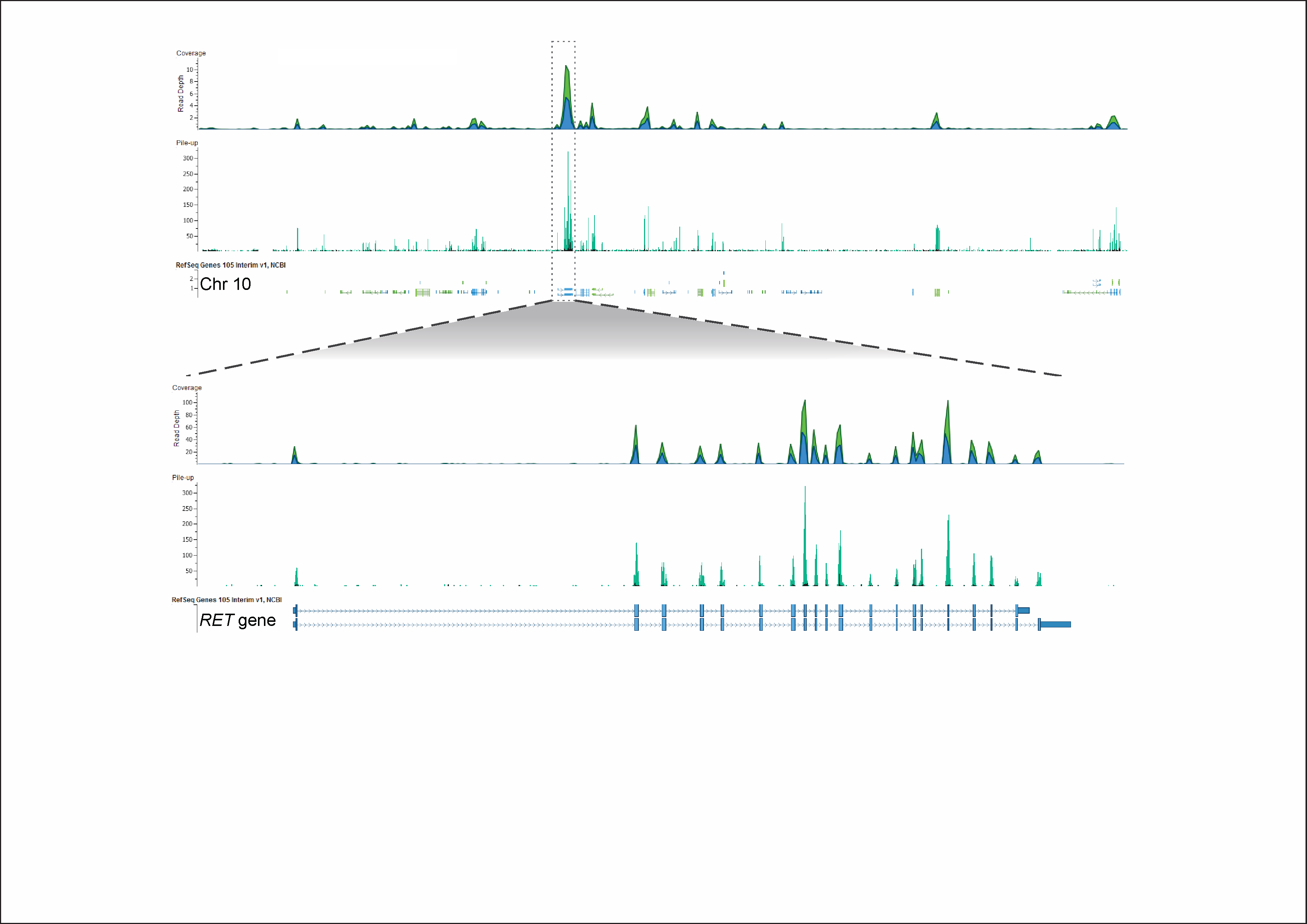

Supplement: Supplementary file 1 — Figure S1. Visual representation of coverage and read alignment of the WES data. The upper panel shows the coverage and read pileup representation of a section of chromosome 10q. It is possible to notice a region with higher number of reads aligned (as delimited by the dotted box) than the regions immediately upstream and downstream of the indicated region. The bottom panel shows a zoom up of the highlighted region, that contains the RET gene. Coverage and read pileup for all exons of the RET gene are depicted. No reads were aligned in introns of RET gene. (TIF 34059 kb) [file 12920_2019_552_MOESM1_ESM.tif]
