# Supplementary material for: Retroposed copies of RET gene: a somatically acquired event in medullary thyroid carcinoma
Source: BMC Med Genomics. 2019 Jul 9;12:104. doi: 10.1186/s12920-019-0552-1 (PMC6617568; doi:10.1186/s12920-019-0552-1)
Supplement: Supplementary file 2 — Table S1. RET analysis in MTC samples used in this study. Table S2. Cell lines used in this study .Table S3. Probe specification for MLPA P169 Hirschsprung-1 assay as designed by MRC-Holland (Amsterdam, The Netherlands). Table S4. Primer design and expected PCR product size for parental copy and Retrocopy. (DOCX 53 kb) [file 12920_2019_552_MOESM2_ESM.docx]

**Supplementary Table 1.** *RET* analysis in MTC samples used in this study

| **Case** | **Sex** | **Disease classification** | **Germline *RET* Mutation** | **Somatic**  ***RET* Mutation** | | **G548V Mutation** |
| --- | --- | --- | --- | --- | --- | --- |
| **1** | M | Sporadic | NEG | | NEG | N |
| **2** | M | Sporadic | NEG | | NEG | N |
| **3** | F | Sporadic | NEG | | NEG | N |
| **4** | M | Sporadic | NEG | | NEG | Y |
| **5** | M | Sporadic | NEG | | NEG | N |
| **6** | F | Sporadic | NEG | | NEG | N |
| **7** | F | Sporadic | NEG | | NEG | N |
| **8** | M | Sporadic | NEG | | NEG | Y |
| **9** | F | Sporadic | NEG | | NEG | N |
| **10** | F | Sporadic | NEG | | NEG | Y |
| **11** | F | Sporadic | NEG | | M918T | Y |
| **12** | M | Sporadic | NEG | | C634Y | N |
| **13** | F | Sporadic | NEG | | NEG | N |
| **14** | M | Sporadic | NEG | | M918T | Y |
| **15** | M | MEN 2A | G533C | | NEG | Y |
| **16** | F | MEN 2A | G533C | | NEG | N |
| **17** | M | MEN 2A | G533C | | NEG | Y |
| **18** | M | MEN 2A | G533C | | NEG | N |
| **19** | M | MEN 2A | G533C | | NEG | Y |
| **20** | M | MEN 2A | G533C | | NEG | N |
| **21** | F | MEN 2A | G533C | | NEG | N |
| **22** | M | MEN 2A | G533C | | NEG | N |
| **23** | F | MEN 2A | C634_ | | NEG | N |
| **24** | F | MEN 2A | C634_ | | NEG | N |
| **25** | F | MEN 2A | C634Y | | NEG | N |
| **26** | M | MEN 2A | C634Y | | NEG | Y |
| **27** | M | MEN 2A | C634Y | | NEG | N |
| **28** | M | MEN 2A | C634Y/Y791F | | NEG | Y |
| **29** | F | MEN 2A | C634Y/Y791F | | NEG | N |
| **30** | M | MEN 2A | C634Y/Y791F | | NEG | N |
| **31** | M | MEN 2A | C634Y/Y791F | | NEG | N |
| **32** | F | MEN 2A | C634Y/Y791F | | NEG | Y |
| **33** | F | MEN 2A | C634Y/Y791F | | NEG | N |
| **34^a^** | M | MEN 2A | G533C | | NEG | N |
| **35^a^** | M | MEN 2B | M918T | | NEG | N |
| **36^a^** | F | MEN 2B | M918T | | NEG | N |
| **37^a^** | F | MEN 2A | C634Y/Y791F | | NEG | N |
| **38^a^** | F | HCC | G533C | | NEG | N |
| **39^a^** | F | HCC | G533C | | NEG | N |

a = fresh-frozen tissue.

**Supplementary Table 2.** Cell lines used in this study

| **Cell line** | **Origem** | **Medium** | **Source** | **Mutation Identified** |
| --- | --- | --- | --- | --- |
| TT | Medullary thyroid carcinoma | F-12K medium supplemented with 10% Fetal Bovine Serum and 100 U/mL penicillin/streptomycin | ATCC Cat. No. CRL-1803 | *RET* C634W |
| TT | Medullary thyroid carcinoma | F-12K medium supplemented with 10% Fetal Bovine Serum and 100 U/mL penicillin/streptomycin | Barry Nelkin, Johns Hopkins University, Baltimore, USA | *RET* C634W |
| MZ-CR-C1 | Medullary thyroid carcinoma | DMEM and Ham’s F12 (1:1 mixture) supplemented with 10% Fetal Bovine Serum and 100 U/mL penicillin/streptomycin | Barry Nelkin, Johns Hopkins University, Baltimore, USA | *RET* M918T |
| XTC.U1 | Hurthle Cell Carcinoma | DMEM and Ham’s F12 (1:1 mixture) supplemented with 10% Fetal Bovine Serum and 100 U/mL penicillin/streptomycin | Ian Ganly, Memorial Sloan Kettering Cancer Center, NY, USA |  |

The authenticity of a panel of human-derived thyroid cancer cell lines was validated through short tandem repeat (STR) profiling to eliminate concerns of cross contamination and were tested for mycoplasma contamination.

**Supplementary Table 3.** Probe specification for MLPA P169 Hirschsprung-1 assay as designed by MRC-Holland (Amsterdam, The Netherlands).

| **Length** | **SALSA MLPA probe** | ***RET* exon** |
| --- | --- | --- |
| 238 | 18027-L23566 | Exon 1 |
| 292 | 18081-L23071 | Exon 2 |
| 196 | 05497-L04920 | Exon 3 |
| 141 | 18025-L06390 | Exon 4 |
| 256 | 05499-L23570 | Exon 5 |
| 202 | 05499-L23570 | Exon 6 |
| 331* | 18331-SP0609 | Exon 7 |
| 373 | 18546-L28617 | Exon 8 |
| 364 | 18084-SP0607 | Exon 9 |
| 389 | 18029-L04927 | Exon 10 |
| 154 | 05505-L05592 | Exon 11 |
| 178 | 05506-L04929 | Exon 12 |
| 220 | 05507-L22379 | Exon 13 |
| 232 | 05508-L20172 | Exon 14 |
| 268 | 05509-L22733 | Exon 15 |
| 469 | 18330-L23098 | Exon 16 |
| 338 | 07288-L22737 | Exon 17 |
| 433 | 18030-L23567 | Exon 18 |
| 183* | 18079-L23061 | Exon 19 |
| 452 | 05514-L23069 | Exon 20 |

***** SNP rs200289472 could influence the 183 nt probe signal (18079-L23061) and SNP rs199572076 could influence the 331 nt probe signal (18331-SP0609-L23070).

**Supplementary Table 4.** Primer design and expected PCR product size for parental copy and Retrocopy

| **Primer**  **Set** | **Region** | **Oligonucleotide (5’ – 3’)** | **Intronless Copy**  **(bp)** | **Parental Copy**  **(bp)** |
| --- | --- | --- | --- | --- |
| ***RET*** |  |  |  |  |
| **1** | Exon 1 | F: TGGCGAAGGCGACGTCCGG | 130 | 23,256 |
|  | Exon 2 | R: GGTCCACATACAGCTTCTCCCAG |  |  |
| **2** | Exon 1 | F: TGGCGAAGGCGACGTCCGG | 921 | 29,170 |
|  | Exon 5 | R: TGATGCAGGTACCACGTCTG |  |  |
| **3** | Exon 1 | F: TGGCGAAGGCGACGTCCGG | 1,347 | 34,031 |
|  | Exon 7 | R: GCAGTTGGCACCAGAGGAA |  |  |
| **4** | Exon 7 | F:TGCCGAACTTCACTACATGG | 2,507 | 15,375 |
|  | Exon 19 | R:GAATCTAGTAAATGCATGGGAAATT |  |  |
| **5** | Exon 10 | F: AGCATTGTTGGGGGACACGA | 1,452 | 13,191 |
|  | Exon 19 | R:GAATCTAGTAAATGCATGGGAAATT |  |  |
| **6** | Exon 14 | F: AAAGTGGGGCCTGGCTAC | 756 | 7,153 |
|  | Exon 19 | R:GAATCTAGTAAATGCATGGGAAATT |  |  |
| **7** | Exon 18 | F:TACCGCCTGATGCTGCAAT | 276 | 1,868 |
|  | Exon 19 | R:GAATCTAGTAAATGCATGGGAAATT |  |  |
| **8** | Exon 7 | F: TGCCGAACTTCACTACATGG | 238 | 1,498 |
|  | Exon 9 | R: GGTGGAGAAGTTCCTGGTGA |  |  |
| ***KRAS*** |  |  |  |  |
|  | Exon 1 | F: GCCTGCTGAAAATGACTGAA | 249 | 18,110 |
|  | Exon 2 | R: AAAGAAAGCCCTCCCCAGTC |  |  |
